# Supplementary material for: Effect of transcranial direct-current stimulation on cognitive function in stroke patients: A systematic review and meta-analysis
Source: PLoS One. 2020 Jun 9;15(6):e0233903. doi: 10.1371/journal.pone.0233903 (PMC7282637; doi:10.1371/journal.pone.0233903)
Supplement: S2 Table — (PDF) [file pone.0233903.s002.pdf]

S2 Table. Search strategy

Medline
1. stroke.mp. or exp Stroke Rehabilitation/ or exp Stroke/
2. stroke.af.
3. poststroke.af.
4. post-stroke.af.
5. 1 or 2 or 3 or 4
6. Cognitive Dysfunction.mp. or exp Cognition Disorders/ or exp Cognitive Dysfunction/ or exp Cognition/
7. exp Cognitive Behavioral Therapy/
8. (cognitive or Cognition).af.
9. 6 or 7 or 8
10. 5 and 9
11. Transcranial Direct Current Stimulation.mp. or exp Transcranial Direct Current Stimulation/
12. 10 and 11
**Embase**
1. stroke.mp. or exp cerebrovascular accident/
2. exp stroke/ or exp stroke rehabilitation/ or exp cerebrovascular disease/
3. Stroke, Lacunar.mp. or exp lacunar stroke/
4. stroke.af.
5. poststroke.af.
6. 1 or 2 or 3 or 4 or 5
7. exp cognitive therapy/ or exp Montreal cognitive assessment/ or Cognitive.mp. or exp cognitive behavioral therapy/ or exp cognitive remediation therapy/ or exp mild cognitive impairment/ or exp cognitive defect/
8. exp cognitive rehabilitation/
9. exp cognition/ or exp cognition assessment/
10. Cognition.af.
11. Cognitive.af.
12. 7 or 8 or 9 or 10 or 11
13. 6 and 12
14. Transcranial Direct Current Stimulation.af.
15. exp transcranial direct current stimulation/
16. tDCS.af.
17. 14 or 15 or 16
18. 13 and 17
**Central**
1. exp Stroke/ or exp Stroke, Lacunar/
2. exp Cerebrovascular Disorders/
3. exp "Recovery of Function"/
4. (stroke or poststroke).af.
5. exp Mild Cognitive Impairment/ or exp Cognitive Dissonance/ or exp Cognitive Therapy/ or exp Cognitive Science/
6. exp Cognition Disorders/
7. exp Cognition/

8. (cognitive or Cognition).af.
9. 1 or 2 or 3 or 4
10. 5 or 6 or 7 or 8
11. (Transcranial Direct Current Stimulation or tDCS).af.
12. 9 and 10 and 11
