# Supplementary material for: Effect of transcranial direct-current stimulation on cognitive function in stroke patients: A systematic review and meta-analysis
Source: PLoS One. 2020 Jun 9;15(6):e0233903. doi: 10.1371/journal.pone.0233903 (PMC7282637; doi:10.1371/journal.pone.0233903)
Supplement: S3 Table — (PDF) [file pone.0233903.s003.pdf]

S3 Table. Risk of bias for included studies judged by Cochrane Collaboration Risk of Bias Tool

Study	Random sequence generation	Allocation concealment	Blinding of participants and personnel	Blinding of outcome assessment	Incomplete outcome data	Selective reporting	Other bias
Gi Jeong Yun	unclear	unclear	low	low	unclear	unclear	unclear
See-Hyun Park	unclear	unclear	low	low	unclear	unclear	unclear
Hussien Ahmed Shaker	unclear	unclear	low	low	unclear	unclear	unclear
Zeng Yaqin	low	unclear	low	unclear	unclear	unclear	unclear
Chen Songlin	low	unclear	high	unclear	unclear	unclear	unclear
Guo Tianlong	unclear	unclear	high	unclear	unclear	unclear	unclear
Jiang Yan	low	unclear	high	unclear	unclear	unclear	unclear
Luo Weihuan	low	unclear	high	unclear	unclear	unclear	unclear
Song Hongyan	low	unclear	high	unclear	unclear	unclear	unclear
Sun Weiming	low	unclear	high	unclear	unclear	unclear	unclear
Tong Jianxia 2018	unclear	unclear	high	unclear	unclear	unclear	unclear
Tong Jianxia 2019	unclear	unclear	high	unclear	unclear	unclear	unclear
Wang Qinjuan	unclear	unclear	high	low	unclear	unclear	unclear
Zheng Jie	low	unclear	high	unclear	unclear	unclear	unclear
Hosseinzadeh	low	low	low	unclear	low	unclear	unclear
