# Supplementary material for: Effect of transcranial direct-current stimulation on cognitive function in stroke patients: A systematic review and meta-analysis
Source: PLoS One. 2020 Jun 9;15(6):e0233903. doi: 10.1371/journal.pone.0233903 (PMC7282637; doi:10.1371/journal.pone.0233903)
Supplement: Attachment — Submitted filename: Response to Reviewers.doc [file pone.0233903.s004.doc]

Dear Editor and Reviewers:

We are truly grateful to yours critical comments and thoughtful suggestions concerning our manuscript entitled “Effect of transcranial direct current stimulation on cognitive function in stroke patients: a systematic review and meta-analysis” (ID: PONE-D-19-28981). We feel lucky that our manuscript went to these reviewers as the valuable comments from them really helped us with the improvement of our manuscript. Based on the comments we received, careful modifications have been made to original manuscript. All changes were marked in red text. In addition, we have consulted native English speakers for paper revision before the submission this time. We hope the new manuscript will meet your magazine’s standard. Below you will find our point-by-point responses to the reviewers’ comments/ questions:

**Reviewer #1:**

**1. Response to comment:** your work examines the effects of anodal tDCS on cognition after stroke. To make this clear to the reader, I suggest to every time referred to “anodal tDCS“ throughout the manuscript, where appropriate. I would also recommend to check for language errors once again.

**Response:** it is really true as reviewer suggested that we should every time referred to the same word throughout the manuscript. However, during the process of revising, we realized that the “active tDCS” are more suitable than “anodal tDCS”, so we have replaced it and also checked the language errors again.

**2. Response to comment:** page nine: the Prospero-ID presented in the abstract is wrong and refers to another publication. Please correct.

**Response:** we are very sorry for our incorrect writing of Prospero-ID, after checking, we have corrected the Prospero-ID as: CRD 42019137191.

**3. Response to comment:** page 9: you state, that tDCS was firstly developed in 1992. Please provide the corresponding citation for that.

**Response:** we are very sorry for our negligence of citing the reference here and we have added a new one now.

**4. Response to comment:** page 11: the term used for describing the comparator “passive stimulus” seems to be a bit ambiguous, here. Apparently, you subsumed no additional intervention and sham tDCS under this term (as can be seen from table 1) and I would suggest to describe this here, also.

**Response:** as reviewer suggested, we should describe the “passive stimulus” more clearly. At this point, we have also realized that the “anodal tDCS” can also lead to ambiguity of using only anodal electrodes without cathodal ones. Hence, in the revised manuscript, we have replaced the “anodal tDCS” with “active tDCS”, and made a explanation for “passive tDCS” in main text as well as table 1.

**5. Response to comment:** page 12: please check the last sentence of the last paragraph in “outcome measures“, there seems to be a word missing.

**Response:** considering the reviewer’s suggestion, we have re-written this sentence.

**6. Response to comment:** page 13: please cite the computer program review manager 5.3, as requested in the programme.

**Response:** as suggested by the reviewer, we have provided a direct link to the review manager 5.3 within the paper.

**7. Response to comment:** page 13: “The publication bias was unable to be assessed due to a small number of studies included.” I would regard this sentence as a part of the discussion section and hereby recommend to move it there.

**Response:** it is really true as reviewer suggested that this sentence should be part of the discussion section. In addition, we found that it is possible and necessary to evaluate publication bias after changing the method of results synthesis(combining the outcomes of MMSE and MoCA). Therefore, we have added the method of publication bias analysis in the subgroup analysis section and showed the result of publication bias in the result section.

**8. Response to comment:** page 13: there seems to be a typo: “we determined that 15 studies with 16 trails“, shouldn‘t it be “trials“ here?

**Response:** we are very sorry for our incorrect writing of “trials”, we’ve corrected it.

**9. Response to comment:** figure 1: you excluded 33 studies, but the reasons are only given for 15. Moreover, you screened 49 full text articles and excluded 33 studies and this resulted in 15 included studies, but 49-33 should be 16. Please clarify. If the difference is due to an inequality of the number of full text publications, I would recommend to explain this to the reader with a comment in the figure caption.

**Response:** we are so sorry for our careless and mistakes in Figure 1. The reasons for excluded studies are not shown completely due to the picture display problem, we have modified the picture. Besides, we missed one excluded study, so we actually excluded 34, leaving 15. Additionally, after this revision, we have removed another study with unavailable data form meta-analysis, finally, 15 studies were included and 13 studies were involved in quantitative synthesis.

**10. Response to comment:** page 14: in the last sentence, after CNT and LOTCA, are these signs, commata? Please check.

**Response:** thank you very much for bringing this to our attention, we carefully reviewed the sentence and decided that it may be inappropriate to place it here, so we have finally deleted this sentence.

**11. Response to comment:** Table 1: in Park 2013 there seems to be a typo (CPR instead of CRP).

**Response:** we apologize for the error of the “CRP” in Table 1 and have corrected.

**12. Response to commen**t: page 17: in the risk of bias section, you stated all trials reported complete outcome data and there were no selective reporting among the studies. I am a bit curious, how you did proof for complete report of outcome data, has there been an a priori published trial protocol for every single included study and you have checked it and there was no deviation? If no, I would disagree with your risk of bias rating and personally would rate it at least as unclear.

**Response:** thank you very much for pointing out the problems in “risk of bias” rating, and we fully accept the reviewer's suggestions. Therefore, we have reassessed the risk of bias: since no priori trial protocol was found, we rated the domains of complete outcome data and selective reporting as unclear.

**13. Response to comment:** Table S3: blinding of participants and personnel - there are only five studies, who used sham tDCS as an adequate method of blinding of participants. I would disagree with your risk of bias rating in the studies which did not use sham tDCS and not rated as high risk of bias in this regard, since this might have introduced superior performance bias because of the absence of a placebo effect. Please check.

**Response:** thank you so much for bringing this to our attention. It is really true as reviewer suggested that the absence of a placebo effect for control groups is easily lead to performance bias. So we have corrected the results in “risk of bias” we have rated these studies without sham tDCS as high risk of bias in terms of blinding of participants and personnel.

**14. Response to comment:** Page 11, Analysis: in order to reduce bias, introduced by analyzing studies with different comparators (sham tDCS versus no sham tDCS) I would recommend to restructure the results section as follows (after having a look at the interventions listed in table 1):

analysis one: active tDCS versus sham tDCS

analysis tool: active tDCS versus no intervention

for reasons of simplicity, you may want to combine results MMSE and MoCA, if you regard both outcome measures measuring the same outcome.

**Response:** thank you very much for these valuable suggestions. According to the reviewer’s suggestion, we have combined the results of MMSE and MoCA and changed the way the results were synthesized, since both of them measure the general cognitive status on a scale of 30 points. Hence, the part of the result has changed quite a bit. In this case, we have performed more subgroup analyses including those based on different comparators (sham tDCS versus no tDCS).

**15. Response to comment:** page 11: in the analysis corresponding to figure 2, you have calculated SMD on the basis of change scores (Park 2013) and total values. However, there is mathematically not allowed. You can have a look into the Cochrane Handbook for further instruction.

**Response:** we are very sorry for our incorrect selection of summary statistics. As reviewer suggested, we looked at the Cochrane Handbook, found that final value and change scores should not be combined together as SMD. After the discussion, we’ve excluded park 2013 from this meta-analysis because no total values was reported in this study.

**16. Response to comment:** Figure 3: in this subgroup analysis, the study “Tong 2018“ suddenly has got a different sample size and different means and standard deviations when compared to the analyses of figure 2, but shouldn‘t it be the same question please check.

**Response:** we are very sorry for our incorrect writing of the sample size, means and standard deviations in original Figure 3. Based on these comments and suggestions, we have made a lot of changes to the result section and also reedited Fig 2 and Fig 3.

**17. Response to comment:** Page 18: “Specialized in attention function“ I would recommend to rename this outcome into “attention“ for “attention function“ or something similar, since otherwise it may be hard for the reader to understand meaning.

**Response:** thank you so much for bringing this to our attention. We have rename this outcome as “Attention performance” according to the reviewer’s suggestion.

**18. Response to comment**: page 19: in the discussion section, you interpret the SMDs of your analyses in units of the underlying outcome measure “The improvement was around 1.01 points for the MMSE and 3.18 points for the MoCA.“ This is wrong. Please interpreted it in units of standard deviations or back transform it to the original outcome measure (the Cochrane Handbook gives advice on this).

**Response:** we are very sorry for our incorrect interpreting of SMDs as a outcome measure. Based on these comments and suggestions, we have carefully revised the results section and re-selected “MD” as outcome measure.

**19. Response to comment:** page 20: last sentence of comparing with others studies - but there is not a univocal corpus of evidence, that tDCS is beneficial… For a comprehensive overview of current reviews on this topic, you may have a look into the published Cochrane reviews of this or a related topic or into the tDCS guideline of Lefaucheur et al. (2017).

**Response:** thank you very much for providing us with these valuable studies. After careful reading, we have re-written the “Comparing with other studies” part according to your suggestions.

**20. Response to comment:** page 20: strengths and limitations: I would add here the threat publication of bias and the comparator issue (sham tDCS versus no intervention), if appropriate.

**Response:** we have made correction according to the reviewer’s comments. Since we have combined the outcome measures of MMSE and MoCA, we added the analysis of publication bias in the revised manuscript. And we have also performed a subgroup analysis based on different comparators (sham tDCS versus no intervention).

**21. Response to comment:** page 21, implications for research: you state, that “intensity (≥1.8 Ma or ≤1.5 Ma)”, but shouldn’t‘t it be “intensity (≥1.5 mA or ≤1.5 mA)” instead? Please check.

**Response:** we are very sorry for our mistake here. The sentence has been deleted because of structural changes to the discussion section.

**Reviewer #2:**

**1. Response to comment:** the reference in the following sentence does not reflect the information contained in authors’ statements that: “Stroke ranks No. 5 among all causes of death and is a leading cause of long-term disability in the world, which produces a major burden to society [1].”

[1] Burke JF, Lisabeth LD, Brown DL, Reeves MJ, Morgenstern LB. Determining stroke's rank as a

cause of death using multicause mortality data. Stroke. 2012;43(8):2207-11.

**Response:** thanks very much for your careful checking of our manuscript. We have revisited the reference and re-written this sentence.

**2. Response to comment:** the authors claim that: “As a neuromodulatory approach, tDCS works by depolarizing or hyperpolarizing neuronal membrane potentials through the activation of sodium- and calcium-dependent channels and NMDA receptor activity, thereby modulating neuronal excitability”. However, no references are provided to support the claim.

**Response:** we are very sorry for our negligence of providing the references here. In terms of the mechanics of tDCS, we referred to these studies:

(1).Lefaucheur JP, Antal A, Ayache SS, Benninger DH, Brunelin J, Cogiamanian F, et al. Evidence-based guidelines on the therapeutic use of transcranial direct current stimulation (tDCS). Clin Neurophysiol. 2017;128(1):56-92.

(2).Paulus W. Transcranial direct current stimulation (tDCS). Suppl Clin Neurophysiol. 2003;56:249-54.

And we have now provided these references in the revised version.

**3. Response to comment:** authors state “…and some preliminary studies also shown beneficial effects of tDCS on cognitive functions both in healthy subjects and stroke patients [9,10]”. However, authors cite:

Webster B, Celnik P, Cohen L. Noninvasive Brain Stimulation in Stroke Rehabilitation.

Fregni F, Boggio PS, Mansur CG, Wagner T, Ferreira MJL, Lima MC, et al. Transcranial direct current stimulation of the…

Please cite specific effects and studies.

**Response:** it is really true as reviewer suggested that we should cite more specific studies here. therefore, we have deleted the original citations and added new ones that focus on the effects of tDCS for healthy or stroke participants.

Additional references are:

(1). Hsu WY, Ku Y, Zanto TP, Gazzaley A. Effects of noninvasive brain stimulation on cognitive

(2). function in healthy aging and Alzheimer's disease: a systematic review and meta-analysis. NEUROBIOL AGING. 2015;36(8):2348-59.

(3). Clemens B, Jung S, Zvyagintsev M, Domahs F, Willmes K. Modulating arithmetic fact retrieval: a single-blind, sham-controlled tDCS study with repeated fMRI measurements. NEUROPSYCHOLOGIA. 2013;51(7):1279-86.

(4). Jo JM, Kim YH, Ko MH, Ohn SH, Joen B, Lee KH. Enhancing the working memory of stroke patients using tDCS. Am J Phys Med Rehabil. 2009;88(5):404-9.

(5). Kazuta T, Takeda K, Osu R, Tanaka S, Oishi A, Kondo K, et al. Transcranial Direct Current Stimulation Improves Audioverbal Memory in Stroke Patients. Am J Phys Med Rehabil. 2017;96(8):565-71.

**4. Response to comment:** on the results section the authors state that: “The results of subgroup analysis suggested that the effectiveness of tDCS seems to be slightly better for ischemic stroke than hemorrhagic stroke, but there was not significant difference.” However, in their conclusions they state: "tDCS is likely to be effective for patients with cognitive impairment after stroke, and the effect might be different among hemorrhagic and ischemic stroke". This conclusion doesn’t seem to be based on facts, at least from their own results.

**Response:** we are very sorry for the inaccurate description of the conclusion. Based on the subgroup analysis, there was actually no statistic difference between ischemic stroke and hemorrhagic stroke. And we have made correction in the conclusion section according to the facts.

**5. Response to comment:** please consider revising the language specifically of the following paragraphs, in order to be able to follow the study properly:

“Except for high morbidity and mortality, the burden of stroke related-disability is another big problem in survivors, of which, the incidence of poststroke cognitive impairment (PSCI) is ranging from 22% to 47% in different studies [3-5], it has had a serious impact on both the economic and the quality of life.”

“Transcranial direct current stimulation (tDCS) was firstly developed in 1992 for

clinical purposes; nowadays, tDCS constitutes as a promising method in neurological

condition regulating.”

“Specifically, some other measurements are used for the evaluation of

attention and memory ability separately, including Computerized Neuropsychological

Test (CNT), Loewenstein occupational therapy cognitive assessment (LOTCA) and

various other forms of testing used in clinical.”

“Seven trials included two stroke types, seven studies focused on ischemic stroke and one studies on hemorrhagic stroke alone.”

**Response:** thanks very much for your careful checking of our manuscript. We have consulted native English speakers for manuscript revision before the submission this time and the above sentences have been revised.
