# Supplementary material for: Effect of transcranial direct-current stimulation on cognitive function in stroke patients: A systematic review and meta-analysis
Source: PLoS One. 2020 Jun 9;15(6):e0233903. doi: 10.1371/journal.pone.0233903 (PMC7282637; doi:10.1371/journal.pone.0233903)
Supplement: Attachment — Submitted filename: Response to Reviewers.docx [file pone.0233903.s005.docx]

**Dear Editor and Reviewers:**

We are truly grateful to yours critical comments and thoughtful suggestions concerning our manuscript entitled “Effect of transcranial direct current stimulation on cognitive function in stroke patients: a systematic review and meta-analysis”( PONE-D-19-28981R1).

Based on the comments we received, careful modifications have been made to original manuscript. And all changes were marked in red text.

We hope the new manuscript will meet your magazine’s standard. Below you will find our point-by-point responses to the reviewers’ comments/ questions:

**Reviewer #1: The authors address most of the comments very well, but a still would like to bring up a few points:**

**Suggestion 1:** -Since you changed the term "anodal tDCS" from the original version of the manuscript to "active tDCS" is now unclear to the reader, which type of tDCS has been provided in the included studies.

I therefore suggest to add the missing information in table 1 (maybe in the "intervention"column), since I regarded as crucial for the reader to exactly know, to what type of tDCS you refer your analysis)

**Our modification:** as suggested by the reviewers, it is important to clarify the type of tDCS included in our analysis. Therefore, we finally decided to use "anodal tDCS" to describe the intervention, just like the original version, since all the included studies used anodal stimulation and it does not lead to ambiguity. (Results section, table 1, page 9-10)

**Suggestion 2:** - you state that "tDCS was first developed for clinical purposes in 1992" [6]. I am not sure, if reference [6] supports this. Maybe the authors would like to refer to PMID 10990547, which is a widely accepted reference in this regard.

**Our modification:** thank you so much for bringing this to our attention. We reviewed the study recommended by the author and cited them in the manuscript. (Introduction section, line 43-44, page 3)

**Suggestion 3:** - - Please cite review manager 5.3 in the following way, which is shown in the programme under "help_>about"

**Our modification:** thank you very much for pointing out the problem. We changed it as “All statistical comparisons were performed in Review Manager 5.3 (http://www.ims.cochrane.org/revman/)[22].”(Materials and methods section, line 118, page 6)

**Suggestion 4:** - @Risk of bias within the studies: Here you refer the corresponding biases, which I regard as discussion and would recommend to move it to the discussion section to "strength and weaknesses"

**Our modification:** as suggested by the reviewer, we moved the “uncertainty about the reporting bias and attrition bias does exist since no priori published trial protocols for the included studies were found.” to the “strength and weaknesses” (Discussion section, line 262-263, page15)

**Suggestion 5:** - @synthesis of results: "Thirteen studies..." I would recommend to also state the number of participants included in these 13 studies.

**Our modification:** as reviewer suggested, we added the number of participants included in these 13 studies.(Results section, line 169, page11)

**Suggestion 6:** - @General cognitive function: you have combined MMSE points together with MoCA points in a mean difference (MD) analysis. As far as I know, this is not allowed, since these are different outcome measures for the same outcome. I therefore recommend to recalculate these analyses and all corresponding subgroup analysis as well as the assessment of publication bias with standardised mean difference (SMD) analysis. I am not sure if this will substantially affect the magnitude and direction of effect, but it would be correct.

**Our modification:** it’s really true as reviewer suggested that we should used the standardized mean differences (SMD) instead of the mean difference (MD) in the general cognitive function analysis, since there are two different outcome measures. We have recalculate these analyses and all corresponding subgroup analysis as well as the assessment of publication bias.

In addition, we added “Sensitivity analysis showed that Song (2019) is a major source of heterogeneity and they did not report the duration of treatment. After removing this study, the SMD for cognitive function test was 1.17 (95% CI 0.83 - 1.51, P < 0.00001, I^2^=78%). For this reason, we omitted this study from the subgroup analyses.”

Then, in the following three subgroup analyses, we excluded the study of Song(2019).

(Result section, line 182-198, page 12)

**Suggestion 7:** -@Subgroup analysis: when reporting the results of subgroup analysis I would recommend to also report the results of the Chi² test for subgroup differences at the very bottom of the forest plots.

**Our modification:** as reviewer suggested, we added the results of the Chi² test and I^2^ value for subgroup differences. (Result section, line 190-198, page 12)

**Suggestion 8:** -@ summary of the evidence: you state, that there were 14 RCTs included, but shouldn't it be either 15 (for qualitative analysis) or 13 (for quantitative synthesis) as presented in figure 1? Please check.

**Our modification:** we are so sorry for our careless and mistakes, we checked the number of included studies and changed the “14 RCTs” to “13 studies” in the manuscript. (Discussion section, line 234, page13)

**Suggestion 8:** - "Furthermore, active tDCS was beneficial for attention specifically but not for memory specifically." I would recommend to move this sentence exactly one sentence downwards in order to make clear, the following sentence about the subgroup analysis refers only to the outcome general cognitive function.

**Our modification:** as suggested by the reviewer, we moved this sentence one sentence downwards.(Discussion section, line 235-238, page14)

**Suggestion 9:** -"In addition, the other two studies excluded from the statistical pooling both reported evidence of effects in favor of active tDCS regarding measures of cognitive function." I would recommend to move this sentence to the results section, because I would regard them as a result, which should first be presented and second be discussed.

**Our modification:** as suggested by the reviewer, we moved this sentence to the result section. (Results section, line 219-220, page 13)

**Suggestion 10:** -@Comparison with other studies: "Moreover, in 2007, the evidence-based tDCS guidelines" shouldn't it be 2017? Please check.

**Our modification:** we are very sorry for our careless and mistakes, we’ve changed it to “2017” in manuscript.(Discussion section, line 250, page 14)

**Suggestion 10:** -@Strengths and limitations: I think a considerable strength of your work is that you have searched specifically Chinese databases and that you have got included several RCTs from China, which the authors of comparable reviews have not. Maybe you would like to state that? I think this also contributes to defect, that source 38 only has identified only one RCT regarding to your research question.

**Our modification:** thank you very much for bringing this to our attention, we added “we conducted an extensive search of the Chinese database, so we obtained several recently published RCTs” and “73% of the included studies came from China and there was a lack of English studies” in the Strengths and limitations part. (Discussion section, line 255-259, page 14-15)

**Suggestion 11:** -@Implications for practice: "are most likely to benefit from transcranial" One of the weaknesses of your work is the risk of bias rating of included studies, meaning that it is likely that your effects may be biased or overestimated. Therefore I would suggest to change, i.e. to downgrade your statement a bit and mention the considerable risk of bias in the included studies

**Our modification:** it is really true as reviewer suggested that we should downgrade the statement, Considering the risk of bias in the included study.

We have modified this part as:

This meta-analysis suggest that stroke patients with cognitive function deficits appear to benefit from transcranial direct current stimulation. However, on the basis of this review of the published literature and considering the risk of bias of included studies, tDCS cannot yet be recommended as a standard therapy for the stokes. More research is needed to determine the potential benefits of tDCS in the future. (Discussion section, line 269-273, page 15)

**Suggestion 12:** -@implications for practice:"(ischemic MD = 3.67; hemorrhagic MD = 3.12) and shorter disease duration (< 30 days MD = 3.68;

30 days -50 days MD =3.10; = 50 days MD = 1.95);" I would recommend not to state the effect sizes here in the discussion section, since I think it will confuse the reader. I think it would be better to describe it and to refer to the corresponding subgroup analysis.

**Our modification:** we rewrote this paragraph due to slight change of the results, and as suggested by the reviewer, we removed the effect sizes in the discussion section and used corresponding subgroups to describe it. (Discussion section, line 280-286, page 15-16)

**Suggestion 13:** -"In addition, we made an effort to divide the results by stimulation parameters, including intensity, the number of sessions and duration" Please move to the results section since these results were not presented before discussion.

**Our modification:** considering the reviewer’s suggestion, we removed the sentences “In addition, we made an effort to divide the results by stimulation parameters, including intensity, the number of sessions and duration. However, no differences were found in these comparisons, possibly due to the heterogeneity of the remaining parameters.” from the discussion section.

However, this subgroup analysis results were not added to the results section, because we find that it is unreasonable to carry out subgroup analysis on only one stimulus parameter without controlling other stimulus variables including intensity, the number of sessions and duration.

**Suggestion 14:** -"Despite the substantial cognitive effects of active" I would downgrade this sentence in regard to the risk of bias issues, is already commented above.

**Our modification:** thank you for reminding us to correct this mistake again, we changed this sentence to “Although current studies have shown that tDCS may be effective on cognitive function recovery after stroke”. (Discussion section, line 291-292, page 17)

**Suggestion 15:** -@Conclusions:What about demanding for more high-quality studies like you did in the abstract? I would support this.

**Our modification:** thank you very much for these valuable suggestions, We revised the last two sentences of the conclusion into: “However, the evidence on the impact of patient characteristics and stimulation parameters is still lacking. More high-quality research is needed to determine the efficacy of tDCS in the treatment of cognitive deficits after stroke and to establish the optimal treatment program.” (Conclusions section, line 303-306, page17)

**Reviewer #2:** I consider that the authors addressed reviewers comments appropriately. The manuscript now is written in a clear language and reflects more accurately their scientific findings.

Special thanks to you for your good comments.
